# Comment on Qiu et al. Effect of Protein-Rich Breakfast on Subsequent Energy Intake and Subjective Appetite in Children and Adolescents: Systematic Review and Meta–Analysis of Randomized Controlled Trials. *Nutrients* 2021, *13*, 2840

**DOI:** 10.3390/nu15071653

**Published:** 2023-03-29

**Authors:** Beate Henschel, Xiwei Chen, Stephanie L. Dickinson, Andrew W. Brown, David B. Allison

**Affiliations:** 1 Department of Epidemiology and Biostatistics, School of Public Health-Bloomington, Indiana University, 1025 E. 7th Street, Bloomington, IN 47405, USA; 2 Arkansas Children’s Research Institute, Little Rock, AR 72202, USA; 3 Department of Biostatistics, University of Arkansas for Medical Sciences, Little Rock, AR 72205, USA

In reading Qiu et al. [1], we found errors that require correction. While researchers may disagree with the inclusion of studies and diets, we retained the study selections from Qiu et al. and focused our corrections on statistical concerns. We address two statistical issues here: (1) not accounting for correlation in cross-over studies, and (2) including multiple diets from the same study without adjustment. Additionally, we noticed data extraction errors. We posted a detailed version of this comment including supporting information at https://osf.io/urb7q/ (files deposited on 23 January 2023).

The first concern is cross-over studies that were included without accounting for correlation within subjects. In cross-over trials, each study participant receives all treatments and serves as their own control. When calculating the treatment effect for a cross-over study, one needs to account for the correlation within subjects [2]. This affects the studies of Baum et al. [3], Bellissimo et al. [4], Kral et al. [5], Leidy & Racki [6], Liu et al. [7], and Mehrabani et al. [8]. We elaborate on our calculations at https://osf.io/urb7q/ (files deposited on 23 January 2023).

The second statistical issue is including multiple treatments from the same study without proper adjustment. Three studies (Bellissimo et al. [4], Kral et al. [5], Mehrabani et al. [5]) included comparisons with more than two treatment or control diets entered as separate studies in the meta-analysis without adjustments. The Cochrane Handbook recommends either combining groups/conditions into a single comparison (recommended), omitting irrelevant groups for the comparison, adjusting sample size in the shared group, or conducting a network meta-analysis [2]. We combined conditions and included one single comparison into our revised meta-analysis (for more details see https://osf.io/urb7q/ (files deposited on 23 January 2023)). 

We reanalyzed the data addressing the issues above and correcting data extraction errors. The overall estimate of the unstandardized mean difference in subsequent energy intake between protein-rich and control breakfasts changed from the reported −111.2 kcal to −106.8 kcal (Table 1). We did not extract and reanalyze the data for the relationship with fullness (Figure 5) and hunger (Figure 6) or other analyses in the paper, but we expect similar issues in all analyses. Although the main conclusion does not change considering the association of breakfast protein content with subsequent energy intake, the errors made by Qiu et al. require formal correction for all analyses. Until corrected, the results and conclusions of the current manuscript are unproven [9]. Other readers who notice the issues outlined above might dismiss the results and conclusions due to incorrect analyses. Correcting those errors may prevent similar errors in future studies (for details see https://osf.io/urb7q/ (files deposited on 23 January 2023)). 

## Figures and Tables

**Table 1:** Associations between protein-rich breakfast and subsequent energy intake (kcal) from a meta-analysis with random effects model.

	Treatment Effect (kcal)	Heterogeneity
As reported in Qiu et al. [1]	−111.2 [−145.42, −76.9]	I^2^ = 67%
Reanalysis: Adjusting for correlation in cross-over studies and combine treatments/control if multiple were listed	−106.8 [−130.3, −83.2]	I^2^ = 51%
